# The role of simple mathematical models in malaria elimination strategy design

**DOI:** 10.1186/1475-2875-8-212

**Published:** 2009-09-14

**Authors:** Lisa J White, Richard J Maude, Wirichada Pongtavornpinyo, Sompob Saralamba, Ricardo Aguas, Thierry Van Effelterre, Nicholas PJ Day, Nicholas J White

**Affiliations:** 1 Centre for Clinical Vaccinology and Tropical Medicine, Nuffield Department of Clinical Medicine, John Radcliffe Hospital, University of Oxford, Oxford, UK; 2 Faculty of Tropical Medicine, Mahidol University, Bangkok, Thailand; 3 Instituto Gulbenkian de Ciência, Oeiras, Portugal; 4 GlaxoSmithKline Biologicals, Rixensart, Belgium

## Abstract

**Background:**

Malaria has recently been identified as a candidate for global eradication. This process will take the form of a series of national eliminations. Key issues must be considered specifically for elimination strategy when compared to the control of disease. Namely the spread of drug resistance, data scarcity and the adverse effects of failed elimination attempts. Mathematical models of various levels of complexity have been produced to consider the control and elimination of malaria infection. If available, detailed data on malaria transmission (such as the vector life cycle and behaviour, human population behaviour, the acquisition and decay of immunity, heterogeneities in transmission intensity, age profiles of clinical and subclinical infection) can be used to populate complex transmission models that can then be used to design control strategy. However, in many malaria countries reliable data are not available and policy must be formed based on information like an estimate of the average parasite prevalence.

**Methods:**

A simple deterministic model, that requires data in the form of a single estimate of parasite prevalence as an input, is developed for the purpose of comparison with other more complex models. The model is designed to include key aspects of malaria transmission and integrated control.

**Results:**

The simple model is shown to have similar short-term dynamic behaviour to three complex models. The model is used to demonstrate the potential of alternative methods of delivery of controls. The adverse effects on clinical infection and spread of resistance are predicted for failed elimination attempts. Since elimination strategies present an increased risk of the spread of drug resistance, the model is used to demonstrate the population level protective effect of multiple controls against this very serious threat.

**Conclusion:**

A simple model structure for the elimination of malaria is suitable for situations where data are sparse yet strategy design requirements are urgent with the caveat that more complex models, populated with new data, would provide more information, especially in the long-term.

## Background

Since the failed campaign to eradicate malaria in the middle of the last century [1], control of malaria infection has been targeted at reduction in morbidity and mortality while protecting the longevity of chemotherapy from drug resistance. Anti-malarial drug resistance occurs when spontaneously arising mutants with reduced drug susceptibility are provided with a survival advantage by the use of the anti-malarial. The sparing use of new anti-malarial drugs has been recommended to minimize the selective pressure on the parasite [2,3]. This is a reasonable argument if the aim is to accept the continued presence of malaria and control its most deleterious effects. However, recent initiatives stating elimination and eventually eradication as the new aim of malaria control require a sea change in strategy [4]. Strategies designed to extend the lifespan of chemotherapies conflict with those likely to be required to successfully and rapidly eliminate the disease [5]. Elimination strategies require that transmission rather than disease is targeted. In this situation, where data are often sparse [6] and the cumulative effect of multiple control methods is complex and dependent on transmission intensity and many other factors, mathematical modelling plays an important role in informing decisions [7].

The phases of eradication of an infectious disease as defined by Molyneux *et al *[8], are as follows:

• Control: Reduction of disease incidence, prevalence, morbidity or mortality to a locally acceptable level as a result of deliberate efforts. Continued intervention measures are required to maintain the reduction.

• Elimination of disease: Reduction to zero of the incidence of a specified disease in a defined geographical area as a result of deliberate efforts. Continued intervention measures are required.

• Elimination of infection: Reduction to zero of the incidence of infection caused by a specified agent in a defined geographical area as a result of deliberate efforts. Continued measures to prevent re-establishment of transmission are required.

• Eradication: Permanent reduction to zero of the worldwide incidence of infection caused by a specific agent as a result of deliberate efforts. Intervention measures are no longer needed.

• Extinction: The specific infectious agent no longer exists in nature or the laboratory

A simple mathematical structure is used to consider the 'elimination of infection' phase. The potential of combining multiple strategies that applied singly would not necessarily result in elimination, but applied in combination have the potential to achieve this aim within the timelines predicted by other more complex modelling exercises [9] is demonstrated. It is predicted that non-treatment control measures (such as deployment of an effective vaccine or insecticide-treated bed nets (ITNs)) could prevent the spread of drug resistance (as suggested in [3]) in a similar way to drug combination therapy, but at the population rather than individual level (as with multiple first-line therapies [10]). It is predicted that in many scenarios even a failure to eliminate the disease would result in a lower cumulative morbidity and mortality than if the attempt were never made. The key exception to this result is the scenario where drug resistance is spreading. Then if the same drugs are used in an elimination strategy, an acceleration of the spread of resistance is predicted, in some cases resulting in higher morbidity following failed elimination attempts.

Eradication of any disease is an ambitious aim that to date has only been achieved for smallpox. There are only a few WHO sanctioned disease targets for eradication or elimination and malaria is not listed among them. Therefore, considering the potential outcomes of failures to eliminate is an important role for mathematical modelling.

## Methods

### Baseline model

This simple compartmental model is based on a previously fully parameterized model for malaria transmission [11] with four compartments with parameters estimated from data sets from areas of diverse transmission intensities ranging from hypo to holoendemic transmission [11]. The model represents a situation where disease is being controlled using treatment of symptomatic malaria and where there may or may not be some reduction of transmission by other means. The model is suitable for all transmission intensities since it includes a clinically immune state that can be maintained through boosting or lost in the absence of exposure. The model outputs include parasite prevalence (as used in [9]) and clinical infection.

### Elimination model

The baseline model is augmented with three examples from the arsenal of interventions targeting malaria transmission that are either currently available or in the final stages of development:

• ITNs and other vector control methods, resulting in a reduction in the force of infection by 30% [12]

• Artemisinin combination therapy.

○ Annual mass screen and treat (MSAT) policy reaching a coverage of 80% of the infected population within three months of each year [5] assuming that the drug will effectively clear blood stage parasites in an average of two weeks' time

• Vaccination using the RTS, S vaccine [13], assuming a vaccine induced reduction in the force of infection by 50% [14] and a duration of immunity of between 1 and 10 years.

○ Given annually to all age groups, reaching a coverage of 80% within a three-month period. The method of administration could be similar to or in conjunction with a MSAT programme.

One plausible intervention that may be widely used in the future is also considered:

• Deployment of the transmission blocking drugs (specifically the gametocidal action of) primaquine or tafenoquine [15].

○ given in combination with every treatment for the duration of the treated infection

○ given annually reaching a coverage of 80% with a duration of effect of two weeks

Full details of both models can be found in Additional file 1.

Elimination is modelled as beginning from a starting scenario, where transmission is being maintained at a fixed level using malaria control and resistance to drug therapy is at a negligible level. To measure the impact of an elimination strategy, the predicted cumulative episodes of clinical malaria are recorded over a 20-year period from the start of the elimination strategy, and are compared with the number of episodes that would arise with a continuation of the control situation. The cumulative proportion of infections that are treated is also recorded as a measure of cumulative selective pressure. The predicted impact of an elimination strategy is the percentage difference between the cumulative episodes of clinical malaria (cumulative person-years of clinical infection averted) or drug pressure, given continued control and given an elimination attempt. Thus positive values of these differences represent a positive impact and negative values represent deleterious effects of elimination strategies.

It is assumed that the potential for the spread of anti-malarial drug resistance is dependent on the selective pressure from treatment and the proportion of infections that are already resistant, which in turn increases with the percentage of resistant infections resulting in an increasing average duration of infection under treatment at the population level. Increasing the average duration of infection would lead to increased transmission, which in turn could lead to more drug treatments and further spread of resistance unless transmission is sufficiently reduced. If transmission is not sufficiently reduced, the process of selective pressure begetting longer durations of infection would eventually render the treatment useless with the population average duration of an infection under treatment approaching that of an untreated infection.

The model assumptions are conservative for drug action by assuming no prophylactic effect of the drug post recovery (see equation for *η *(*t*) in Additional File 1). This is a reasonable assumption for artemisinin or quinine monotherapies because of the rapid elimination kinetics of the drug, and is also reasonable for artemisinin combination therapy (ACT) in low transmission settings since individuals are unlikely to be exposed to malaria infection during the period of "post-treatment prophylaxis" of the partner drug. However, this prophylactic effect could be significant in high transmission areas and act to increase the speed of elimination and also the spread of resistance. It is assumed that during MSAT, individuals do not need to seek treatment for clinical infections since their infections would be detected and treated during MSAT at a suitably high coverage (80%). This is also a conservative assumption.

Since the models are deterministic, the prevalence of malaria will not reduce to a value of exactly zero. Elimination is therefore defined as having been achieved when the parasite prevalence has reduced to less than 0.001% and the rate of change of parasite prevalence thereafter is negative. Other modelling work has demonstrated that definitions of this type in deterministic models are consistent with their stochastic counterparts (see [5] for example). A different threshold will alter the predicted time to elimination by a few years but not the qualitative behaviour of the model.

A user-friendly web-based version of the elimination model is currently under development [16].

## Results and Discussion

**Figure 1:**
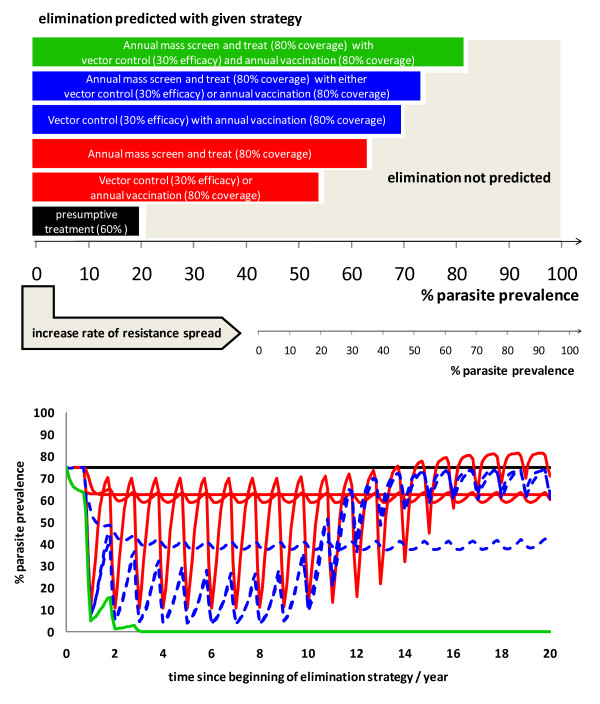
**Top: Diagram illustrating the % parasite prevalence thresholds under which elimination is predicted for single and combinations of interventions**. The large scale represents settings where drug resistance is not spreading and the small scale below represents settings where drug resistance is already spreading. Bottom: A graph showing the percentage parasite prevalence over time for all combinations of up to three currently available control measures (increased vector control; mass screen and treat; annual mass vaccination) compared with treatment of clinical infection only (solid black line). Single measures (solid red) are compared with two combined measures (dashed blue) and three combined measures (solid green).

As Figure 1 illustrates, in the absence of drug resistance, for low transmission intensities (up to about 20% parasite prevalence) it is predicted that presumptive treatment of clinical cases with a sufficiently high coverage (60%) is adequate for elimination. For intermediate transmission intensities (from 20% to 50% parasite prevalence), the model predicts that the introduction of any one of MSAT, ITN or annual vaccination, at the levels described earlier, would be adequate to eliminate malaria infection. For intermediate to high transmission intensities (from 50% to 75% parasite prevalence), any two combined interventions at least would be required for elimination of malaria infection. For very high levels of transmission (parasite prevalence from 75% to 85%) three combined interventions are predicted to be required to eliminate malaria infection. Resistance spreading at a given speed is predicted to have the effect of decreasing the prevalence thresholds for elimination for each strategy, rendering some strategies ineffective in any setting.

For a high transmission setting (75% parasite prevalence with 10% of infections being clinically apparent, Figure 1) into which a single intervention (red solid), pairs of interventions (blue dashed), or a combination of all three (green solid) are introduced, only a combination of all three interventions was sufficient to reduce the predicted reproduction number below unity, thus predicting that elimination would be possible.

### Impact of elimination strategies

Unless otherwise stated, conditions are varied using a starting point of the three-intervention combined elimination strategy (green line in Figure 1).

**Figure 2:**
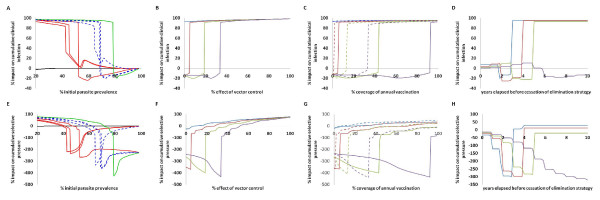
**Graphs showing the percentage impact of specific strategies on cumulative clinical cases (top row) and cumulative drug pressure (bottom row)**. In graphs A and E, the pre-intervention parasite prevalence was varied for all the strategies used to produce Figure 1, the colour scheme being the same. In the remaining graphs (B-D and F-H) four transmission settings were considered given pre-intervention parasite prevalences of: 65% (light blue); 70% (dark red); 75% (light green); 80% (purple). In graphs B and F the percentage effect on the force of infection of vector control was varied and modelled in combination with mass-vaccination and MSAT at the levels used to produce the green line in Figure 1. In graphs C and G, the level of coverage of annual vaccination was varied for a vaccine with duration of 1 (solid lines) and 10 (dashed lines) years and modelled in combination with vector control and MSAT at the levels used to produce the green line in Figure 1. In graphs D and H, the number of years since the beginning of the elimination strategy (see figure 1) until a reversion to the original control strategy is varied.

If the pre-intervention parasite prevalence is varied from 20% to 98% (Figure 2A and 2E), then the predicted impact on clinical infection of strategies not including MSAT is positive for all transmission settings. When MSAT is included, the predicted impact can be negative. This negative impact arises from resistance spread being accelerated by MSAT to the point where the elimination strategy fails. In lower transmission settings, during successful elimination campaigns, the selective pressure is predicted to be positive, even for strategies including MSAT, meaning that there is less selective pressure from the campaign compared to standard presumptive treatment. For higher transmission settings, the impact on selective pressure is negative and is significantly lower when elimination fails.

Increasing vector control efficacy levels and vaccination coverage levels have similar effects (Figure 2B, F, C and 2G). Unsuccessful elimination strategies are predicted to have a negative impact on clinical infections (again only if drug resistance is spreading) (Figure 2B and 2D). For levels above those required for elimination, both vector control efficacy and annual vaccination coverage have threshold levels above which the impact on selective pressure is positive (Figure 2F and 2G), predicting that the use of these interventions at high enough coverage in combination with MSAT will act to prevent or even reverse the spread of resistance during the campaign.

Cessation of a strategy before elimination is achieved predicts a cumulative decrease in clinical infection in the absence of the spread of resistance. If resistance is spreading, then the early cessation of the strategy is predicted to result in more clinical infection due to the acceleration of resistance spread by the MSAT component of the strategy (Figure 2D and 2H).

If a single dose of a transmission blocking drug is assumed as resulting in a 90% reduction in infectiousness lasting two weeks, then results indicate that an annual MSAT would not be effective whereas continuous administration, in combination with all treated cases for the duration of infection, has a similar effect as an annual mass-vaccination strategy. The action of a transmission-blocking drug is assumed to be orthogonal to the other three approaches. That is, that the transmission blocking effects are assumed independent of the effects of the other controls with no redundancy.

### Comparison with other models

In this section, the simple model developed here is compared with specific examples from three more complex models using similar input parameters to demonstrate the ability of the simple elimination model to reproduce the qualitative behaviour of more complex models.

#### An individual-based model with MSAT and vector control

**Figure 3:**
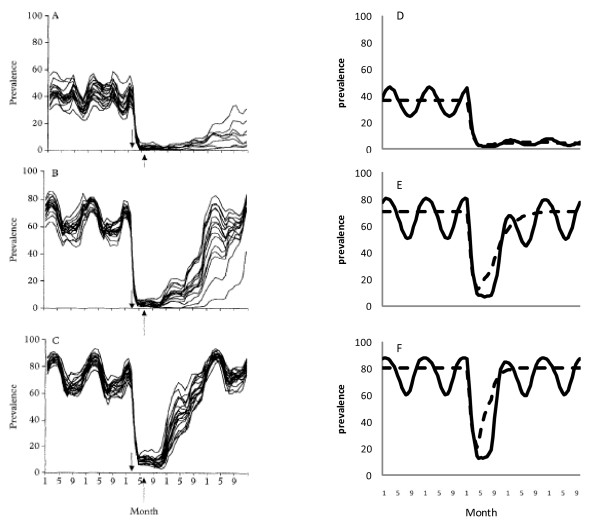
**Graphs comparing predictions from a complex model (graphs A to C copied from graphs A to C, Figure Three in **[17]**) and the simple elimination model with (solid) and without (dashed) seasonal forcing (graphs D to F) for the effect on parasite prevalence of a double intervention of MSAT and increased bed net usage for a range of transmission intensities**.

This is a stochastic individual-based model [17] that combines MSAT reaching 80% in three months in February and ITNs started in June with 30% reduction in transmission and seasonal transmission (amplitude of transmission 50%). The model is presented in the original publication for three different transmission settings (with pre-intervention parasite prevalences of about 40%, 70% and 80%). The simple model can replicate the general behaviour with the same input parameters for all three transmission settings (Figure 3). Seasonality has the effect of enhancing the post-intervention nadir of parasite prevalence in high transmission settings.

#### An individual-based model to predict the impact of vaccination

**Figure 4:**
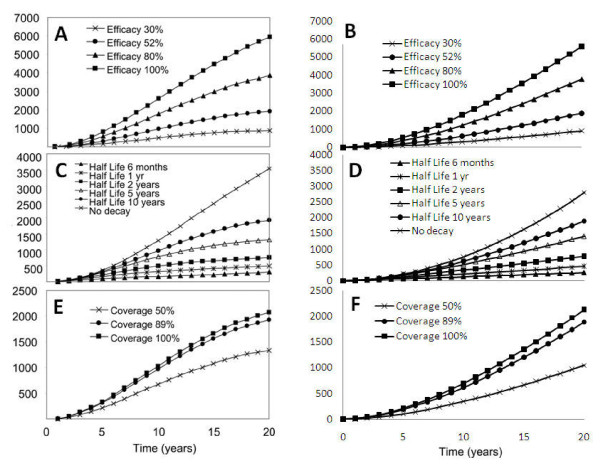
**Graphs comparing predictions from a complex model (graph A copied from graph a, Figure Four in **[18]**; graph C copied from graph a, Figure Six in **[18]**; graph E copied from graph a, Figure Seven in **[18]**) and the simple elimination model (graphs B, D and F) for the effect of vaccination at birth on cumulative incidence of all malaria infections at various efficacies, with various vaccine half-lives and at various coverage levels respectively**.

This is an individual based model [18] that assumes a coverage of 89% with a protective effect against infection with initial values of 30%, 55%, 80% and 100% that decrease in value with a half life of 10 years. The simple model assumes fixed (i.e. not waning) effects of 30%, 55%, 80% and 100% with an average duration to give a half-life of 10 years with a pre-intervention parasite prevalence of 58% and coverage of 89%. The pre-intervention prevalence of 58% was chosen as suitable to reflect the prevalence distribution with age, shown in Figure 2A of [18]. The simpler model does not capture the subtle transient dynamics (such as the plateau in time) or within-host dynamics, but does predict similar values and behaviour in the short term (Figure 4).

#### A multi-strain model to consider elimination in the presence of significant drug resistance

**Figure 5:**
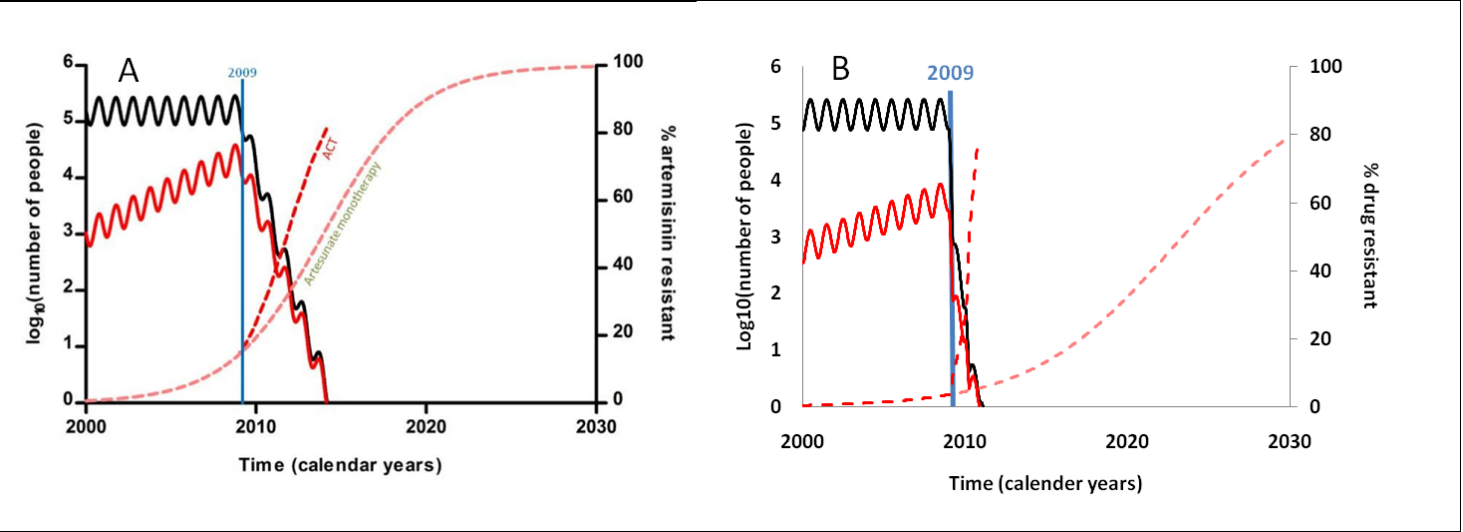
**Graphs comparing predictions from a complex model (graph A copied from Figure Three in **[5]**) and the simple elimination model (graph B) for the dynamics of sensitive and resistant parasite prevalence during an annual MSAT elimination strategy over 20 years**.

Deterministic and stochastic time-series models that explicitly model the dynamics of drug usage and decay with transmission of sensitive and resistant strains of the parasite [5]. Both models show that an elimination strategy using anti-malarial drugs will precipitate a more rapid spread of resistance than typical control (Figure 5). Both models also warn against the early cessation of such a strategy (see Figure 2, graph D).

## Conclusion

With the proliferation of partially effective interventions proposed for the elimination of malaria, informed choices are needed now on how they should be used. Simple compartmental modelling allows the effects of large number of possible combinations of controls and administration methods to be predicted across a range of transmission settings. The model presented here is deliberately simple in nature. It is for the purposes of understanding the more general behaviour of malaria transmission specifically during elimination and could be used as a first step by policy makers for strategy planning for a few years ahead. The benefit of a simple model is that it requires very little data input and can be run quickly for a range of scenarios. For longer term planning, more complex models are required. The benefit of such models is that they are likely to predict accurately for a longer time period, but only if they are correctly parameterized with large and multiple datasets for the specific country. If these data are not available an alternative approach is to use simple models initially and then upgrade them with increasing complexity as more data are collected during the elimination process. This approach has been applied successfully for other diseases in the UK by the Health Protection Agency [19].

A simple mathematical model is used here to demonstrate the interrelationships between different interventions and the potential for elimination when they are combined. The qualitative behaviour corresponds well to that of three more complex models that consider different aspects of malaria transmission in far more detail. Comprehensive models that consider every aspect of malaria transmission and infection are highly computer intensive, often sensitive to poorly characterized input parameters, and the time required to run all combinations of potential interventions could be longer than the time available to contribute to elimination policies within a realistic timeframe. The number of combinations of interventions is large. Simple models that can consider many scenarios in a short length of time are useful in reducing the number of runs required for the more complex but computer intensive models tailored to specific regions. The ability of this simple model to reproduce the main trends predicted by its more complex counterparts is demonstrated indicating its value as a precursor to more complex models to allow predominant trends and behaviours to be explored very quickly before quantitative predictions and recommendations are retrieved.

The model includes one interaction between interventions as an example, namely that when MSAT is active we assume that presumptive treatment does not occur. Other interactions that may negatively affect elimination (such as the possibility that individuals who experience high biting rates also experience a lower coverage from vaccination) have not been considered. By definition, a simple model does not include everything. However the simple model structure could easily be extended to include such an effect if it were considered significant for a particular setting. For this example, individuals not covered by vaccination could be given a higher transmission coefficient during the intervention.

If resistance is not spreading, predictions for impact on clinical infection are positive even when elimination fails. This is because, in the absence of an elimination strategy, transmission and clinical infection will continue at the same level, resulting in an accumulation of cases of clinical infection. An elimination strategy is likely to involve aggressive treatment of infected individuals regardless of their clinical signs. Early cessation of an aggressive elimination strategy in a high transmission setting and the resulting rebound in clinical infection (due to increased transmission in a population with reduced immunity) was considered. This is predicted to have a deleterious effect only in the presence of the spread of resistance. Even some successful elimination strategies will result in higher selective pressure than control. However, in combination with other interventions, such as vector control or vaccination, the deleterious effects can be abated and in some cases removed completely. This analysis provides evidence for the argument that attempting malaria elimination using a suitable strategy is low risk in terms of the burden of clinical disease and the spread of drug resistance.

The model presented here can also be used for the initial stages of target product profiling in terms of the target efficacy levels and durations required for a given product applied in combination with of a range of interventions for a range of transmission settings. The model can be used to propose suitable delivery strategies of new and existing products and to assess the most likely relative efficacy of such products.

In conclusion, the modelling exercise demonstrates that combining interventions with suitable delivery strategies can protect drugs from selective pressure through indirect effects on the transmission dynamics. Also, single interventions that have previously been assessed as weak can be combined to make a seemingly unhopeful strategy more likely to succeed due to the herd effect of combining strategies. This is a conclusion that reiterates the results of previous modelling work [20-22]. Furthermore, in the current climate of increased commitment to the eradication of malaria, the model results indicate that even in a high transmission setting a suitable combination of interventions could achieve elimination through a sustained aggressive integrated control strategy without significantly increasing selective pressure against anti-malarial drugs.

## Abbreviations

ITN: insecticide-treated bed net; WHO: World Health Organization; MAST: mass screen and treat; ACT: artemisinin combination therapy.

## Competing interests

TE is an employee of GlaxoSmithKline Biologicals.

## Authors' contributions

LW developed the model structures (based on previous work by RA, LW *et al*.) and performed the mathematical analysis. RM, WP, SS, TE, and RA participated in developing the initial concept of the study, review of the mathematical analysis and manuscript revision. RM, ND and NW provided the clinical context of the study and participated in manuscript revision. All authors read and approved the final manuscript.

## Supplementary Material

Additional file 1 **Mathematical models.** Click here for file
