# Supplementary material for: The role of simple mathematical models in malaria elimination strategy design
Source: Malar J. 2009 Sep 14;8:212. doi: 10.1186/1475-2875-8-212 (PMC2754494; doi:10.1186/1475-2875-8-212)
Supplement: Additional file 1 — Mathematical models. [file 1475-2875-8-212-S1.pdf]

The Baseline and Elimination Model Equations, Derivations and Parameter Values

Lisa J. White[1,2*], Richard J Maude[1,2], Wirichada Pongtavornpinyo[2], Sompob Saralamba[2], Ricardo Aguas[3], Thierry Van Effelterre[4], Nicholas PJ Day[1,2], Nicholas J White[1,2]

1. Centre for Clinical Vaccinology and Tropical Medicine, Nuffield Department of Clinical Medicine, John Radcliffe Hospital, Unversity of Oxford, Oxford, UK
2. Faculty of Tropical Medicine, Mahidol University, Bangkok, Thailand
3. Instituto Gulbenkian de Ciência, Oeiras, Portugal
4. GlaxoSmithKline Biologicals, Rixensart, Belgium

* Corresponding author

Baseline model

The diagram shows the compartmental structure of the model with time dependent variables: S (uninfected and non-immune); I_1 (infected with no prior immunity); R (uninfected with immunity); I_2 (infected with prior immunity). Model output variables are: C (clinical infections); P (proportion of people receiving treatment or selective pressure); C_c (cumulative clinical infections); P_c (cumulative selective pressure).

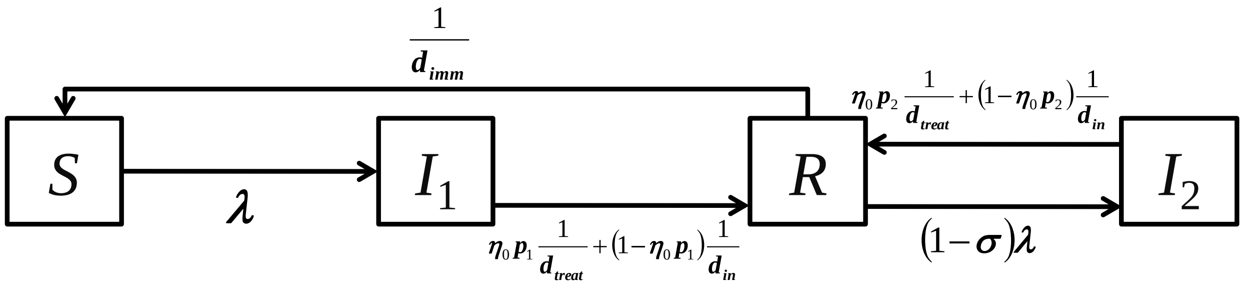

The equations below describe this system:

$$S' = \frac{N}{L} - \left(\lambda + \frac{1}{L}\right)S + \frac{1}{d_{imm}}R$$

$$I_1' = \lambda S - \left(\eta_0 p_1 \frac{1}{d_{treat}} + (1-\eta_0 p_1)\frac{1}{d_{in}} + \frac{1}{L}\right)I_1$$

$$I_2' = \lambda R - \left(\eta_0 p_2 \frac{1}{d_{treat}} + (1-\eta_0 p_2)\frac{1}{d_{in}} + \frac{1}{L}\right)I_2$$

$$R' = \left(\eta_0 p_1 \frac{1}{d_{treat}} + (1-\eta_0 p_1)\frac{1}{d_{in}}\right)I_1 + \left(\eta_0 p_2 \frac{1}{d_{treat}} + (1-\eta_0 p_2)\frac{1}{d_{in}}\right)I_2 - \left[\lambda + \frac{1}{d_{imm}} + \frac{1}{L}\right]R$$

$$\lambda = R_0\left(\frac{1}{L} + \frac{1}{d_{treat}}\right)\frac{(I_1 + I_2)}{N}$$

$$C = p_1 I_1 + p_2 I_2$$

$$P = \frac{\eta_0 (p_1 I_1 + p_2 I_2)}{I_1 + I_2}$$

$$C_c' = C$$

$$P_c' = P$$

$$p_{res} = \frac{\frac{1}{d_{treat}} - \frac{1}{d_{treat0}}}{\frac{1}{d_{in}} - \frac{1}{d_{treat0}}}$$

$$\tau' = -kPp_{res}\left(\tau - \frac{1}{d_{in}}\right)$$

$$d_{treat} = \frac{1}{\tau}$$

The initial conditions of the variables S, I_1, I_2 and R are given by the equilibrium solution of the system with $d_{treat} = d_{treat0}$. The initial conditions of C_c and P_c are zero and the initial condition of τ is $(1/d_{treat0})$.

Parameter values

Symbol	Definition	Value	Source
N	Population size	10^6	
L	Average life expectancy	50 years	
d_{imm}	Average duration of immunity in the absence of re-exposure	1 year	[1]
d_{in}	Average duration of untreated infection	0.5 year	Assumed
d_{treat0}	Average duration of treated sensitive infection	2 wks (figure 5)<br>4 wks (other figures)	[2]<br>Assumed
p_1	Average proportion of infected individuals without preexisting immunity that have clinical malaria	0.87	[1] & $p_1 = \frac{d_{treat}(d_{in} - 14.12)}{d_{treat} d_{in} - \eta_0}$
p_2	Average proportion of infected individuals that have clinical malaria who have experienced infection previously within a year	0.08	[1] & $p_1 = \frac{d_{treat}(d_{in} - 2.23)}{d_{treat} d_{in} - \eta_0}$
k	coefficient of speed of spread of resistance	0 - 0.1	Assumed
η_0	precentage of individuals with clinical infection that receive treatment	60	Assumed
R_0	Basic reproduction number	2.4 – 63	corresponds to parasite prevalence of 20% to 98%

Given presumptive treatment of clinical cases at a coverage of 60%, k=0.04 indicates that the percentage of resistant infections would increase from 0% to about 50% in 20 to 40 years depending on transmission intensity. k=0.08 indicates that the percentage of resistant infections would increase from 0% to about 50% in 5 to 20 years depending on transmission intensity

Elimination model

This model is produced from two linked copies of the basic model with the presence or absence of the subscript *V* representing individuals who have received or not received effective vaccine respectively. The additional variables *h*, *m* and *x* represent respectively rates of receiving routine treatment, receiving treatment as part of MSAT and vaccination.

$$S' = (1-c_b)\frac{N}{L} - \left(\pi\lambda + \frac{1}{L}\right)S + \frac{1}{d_{imm}}R - x(t)S + yS_V$$

$$I_1{}' = \pi\lambda S - \left(\frac{(\eta p_1 + m)}{d_{treat}} + \frac{(1-\eta p_1 - m)}{d_{in}} + \frac{1}{L} + \frac{m}{d_{treat}}\right)I_1 - x(t)I_1 + yI_{1V} - m(t)I_1$$

$$I_2{}' = \pi\lambda R - \left(\frac{(\eta p_2 + m)}{d_{treat}} + \frac{(1-\eta p_2 - m)}{d_{in}} + \frac{1}{L} + \frac{m}{d_{treat}}\right)I_2 - x(t)I_2 + yI_{2V} - m(t)I_2$$

$$R' = \left(\frac{(\eta p_1 + m)}{d_{treat}} + \frac{(1-\eta p_1 - m)}{d_{in}} + \frac{m}{d_{treat}}\right)I_1 + \left(\frac{(\eta p_2 + m)}{d_{treat}} + \frac{(1-\eta p_2 - m)}{d_{in}} + \frac{m}{d_{treat}}\right)I_2$$
$$-\left[\pi\lambda + \frac{1}{d_{imm}} + \frac{1}{L}\right]R - x(t)R + yR_V$$

$$S_V' = c_b\frac{N}{L} - \left(\rho\pi\lambda + \frac{1}{L}\right)S_V + \frac{1}{d_{imm}}R_V + x(t)S - yS_V$$

$$I_{1V}{}' = \rho\pi\lambda S_V - \left(\frac{(\eta p_1 + m)}{d_{treat}} + \frac{(1-\eta p_1 - m)}{d_{in}} + \frac{1}{L} + \frac{m}{d_{treat}}\right)I_{1V} + x(t)I_1 - yI_{1V}$$

$$I_{2V}{}' = \rho\pi\lambda R_V - \left(\frac{(\eta p_2 + m)}{d_{treat}} + \frac{(1-\eta p_2 - m)}{d_{in}} + \frac{1}{L} + \frac{m}{d_{treat}}\right)I_{2V} + x(t)I_2 - yI_{2V}$$

$$R_V' = \left(\frac{(\eta p_1 + m)}{d_{treat}} + \frac{(1-\eta p_1 - m)}{d_{in}} + \frac{1}{L} + \frac{m}{d_{treat}}\right)I_{1V} + \left(\frac{(\eta p_2 + m)}{d_{treat}} + \frac{(1-\eta p_2 - m)}{d_{in}} + \frac{1}{L} + \frac{m}{d_{treat}}\right)I$$
$${}_{2V} - \left[\rho\pi\lambda + \frac{1}{d_{imm}} + \frac{1}{L}\right]R_V + x(t)R - yR_V$$

$$\lambda = \frac{R_0\left(\frac{1}{L} + \frac{1}{d_{treat}}\right)(I_1 + I_2 + I_{1V} + I_{2V})}{N}$$

$$C = p_1(I_1 + I_{1V}) + p_2(I_2 + I_{2v})$$

$$P = \frac{\eta p_1(I_1 + I_{1V}) + \eta p_2(I_1 + I_{1V}) + m(I_1 + I_{1V} + I_2 + I_{2V})}{(I_1 + I_{1V} + I_2 + I_{2V})}$$

$$C_c' = C \qquad \eta(t) = \begin{cases} 0 & \textbf{for } \mathrm{mod}(t,1) \ge \frac{9}{12} \\ \eta_0 & \textbf{for } \mathrm{mod}(t,1) < \frac{9}{12} \end{cases}$$

$$P_c' = P$$

$$p_{res} = \frac{\frac{1}{d_{treat}} - \frac{1}{d_{treat0}}}{\frac{1}{d_{in}} - \frac{1}{d_{treat0}}} \qquad x(t) = \begin{cases} -4\ln\left(\frac{100-c}{100}\right) & \textbf{for } \mathrm{mod}(t,1) \ge \frac{9}{12} \\ 0 & \textbf{for } \mathrm{mod}(t,1) < \frac{9}{12} \end{cases}$$

$$\tau' = -kP\,p_{res}\left(\tau - \frac{1}{d_{in}}\right) \qquad m(t) = \begin{cases} 1 - \exp\left(4\ln(1 - \mathrm{m_{sat}})\mathrm{mod}\left(t - \frac{9}{12}\right)\right) & \textbf{for } \mathrm{mod}(t,1) \ge \frac{9}{12} \\ 0 & \textbf{for } \mathrm{mod}(t,1) < \frac{9}{12} \end{cases}$$

$$d_{treat} = \frac{1}{\tau}$$

The initial conditions are as for the baseline model for the non-vaccine variables. The initial conditions for the vaccine variables are all zero. To derive the formula for the rate of vaccination $x(t)$, we consider a population being vaccinated at a constant rate q given an initial condition of 100%. Then the differential equation describing the percentage of vaccinated individuals, n, is given by $\boldsymbol{n}' = \boldsymbol{q}(100 - \boldsymbol{n})$ with solution $\boldsymbol{n}(\boldsymbol{t}) = 100 - 100\boldsymbol{e}^{-\boldsymbol{qt}}$. Thus to achieve a coverage, c, in 3 months (i.e. ¼ years) $\boldsymbol{n}\left(\frac{1}{4}\right) = 100 - 100\boldsymbol{e}^{-\frac{\boldsymbol{q}}{4}} = \boldsymbol{c}$, thus $\boldsymbol{q} = -4\ln\left(\frac{100 - \boldsymbol{c}}{100}\right)$, which should be the function for $x(t)$ during the period of vaccination each year.

Parameter values

Symbol	Definition	Value	Source
ρ	proportional vaccine efficacy	0 to 1	[3]
π	reduction in force of infection due to increased vector control	0.3	[4]
c	annual vaccine coverage	80 %	
y	rate of loss of vaccine effect	1- 10 year^{-1}	[3]
m_{sat}	proportion of infected people treated during MSAT	0.8	[2]

<u>References</u>

1. R Aguas, LJ White, RW Snow, MG Gomes: **Prospects for malaria eradication in sub-Saharan Africa**. *PLoS ONE* 2008, **3**:e1767.
2. RJ Maude, W Pontavornpinyo, S Saralamba, R Aguas, S Yeung, AM Dondorp, NP Day, NJ White, LJ White: **The last man standing is the most resistant: eliminating artemisinin-resistant malaria in Cambodia**. *Malaria Journal* 2009, **8**:31.
3. N Maire, JJ Aponte, A Ross, R Thompson, P Alonso, J Utzinger, M Tanner, T Smith: **Modeling a field trial of the RTS,S/AS02A malaria vaccine**. *American Journal of Tropical Medicine and Hygiene* 2006, **75**:104-10.
4. T Sochantha, S Hewitt, C Nguon, L Okell, N Alexander, S Yeung, H Vannara, M Rowland, D Socheat: **Insecticide-treated bednets for the prevention of Plasmodium falciparum malaria in Cambodia: a cluster-randomized trial**. *Tropical Medicine and International Health* 2006, **11**:1166-77.
